# Evaluation of Sella Turcica Bridging and Morphology in Different Types of Cleft Patients

**DOI:** 10.3389/fcell.2020.00656

**Published:** 2020-07-22

**Authors:** Mohammad Khursheed Alam, Ahmed Ali Alfawzan

**Affiliations:** ^1^Orthodontic Division, Department of Preventive Dental Science, College of Dentistry, Jouf University, Sakaka, Saudi Arabia; ^2^Department of Preventive Dentistry, College of Dentistry in Ar Rass, Qassim University, Ar Rass, Saudi Arabia

**Keywords:** sella turcica, sella turcica bridging, morphometry, bilateral cleft lip and palate, unilateral cleft lip and palate

## Abstract

**Objectives:**

To evaluate sella turcica (ST) bridging, associated anomalies, and morphology, in subjects with four different types of clefts, and compare them with non-cleft (NC) subjects.

**Materials and Methods:**

A total of 123 (31 NC and 92 cleft) Saudi subjects who had their lateral cephalogram (Late. Ceph.), orthopantomogram (OPG), and clinical details for ordinary diagnosis were included in the study. Among 92 cleft subjects, 29 had bilateral cleft lip and palate (BCLP), 41 had unilateral cleft lip and palate (UCLP), nine had unilateral cleft lip and alveolus (UCLA), and 13 with unilateral cleft lip (UCL). ST bridging and seven parameters related to ST morphology and skeletal malocclusion were analyzed using Late. Ceph. Associated dental anomalies in ST bridging subjects were investigated using OPG. The images were investigated using artificial intelligence driven Webceph software. Multiple statistical tests were applied to see the differences between gender and among cleft vs NC subjects.

**Results:**

ST bridging was found to be higher in cleft subjects (22.82%). Most of the cleft subjects had severe skeletal Class III malocclusion associated with multiple types of dental anomalies (impacted canines, congenital missing, and presence of supernumerary teeth). No significant gender disparities in all seven parameters of ST morphology were found between NC and cleft groups. However, there were significant differences when compared among four different types of cleft individuals vs NC subjects.

**Conclusion:**

ST bridging is more prevalent in cleft subjects along with Class III malocclusion and associated dental anomalies. ST morphometry differs significantly between cleft vs NC subjects. BCLP exhibits smaller values of all seven parameters as compared to all other groups.

## Introduction

Lateral cephalogram (Late. Ceph.) uses a number of landmarks as reference points for analysis/study of craniofacial structures. Sella turcica (ST) serves as one such important landmark in the cranium on Late. Ceph. The sella point or the center of the ST is a point in the cranial base which is situated at the midpoint of ST that accommodates the pituitary gland (Celik-Karatas et al., 2015). It plays an important role in cephalometric analysis and helps us identify pathologies related to pituitary gland and hence becomes an exceptional source of information, specifically those syndromes that affect craniofacial region. A thorough knowledge of its radiological anatomy and variations may help us evaluate the growth and recognize any deviation in a variety of anomalies or pathological situations, and the possible outcome of the orthodontic treatment in such situations.

Congenital anomalies, though identified at birth often, get initiated during pregnancy due to chromosomal abnormalities. A gamut of congenital anomalies occurs in the craniofacial region, cleft lip and palate (CLP) being the most common anomaly in the head and neck region, only second to congenital heart disease in the whole body. Hence, cleft deformities have been included in their Global Burden of Disease initiative, by World Health Organization (WHO). CLP is quite variable in its presentation and affects about 1.17/1000 birth overall 1.30 of every 1000 live births in Saudi (Sabbagh et al., 2015) and Asian populations (Cooper et al., 2006). CLP has a multifactorial etiology with genetics and environmental factors to be the major contributing factors (Mars and Houston, 1990). The clefts have been classified depending upon the extent of involvement and their location as cleft palate, cleft lip, unilateral cleft lip (UCL), unilateral cleft lip and alveolus (UCLA), unilateral cleft lip and palate (UCLP), bilateral cleft lip and palate (BCLP), etc. The affected children may have retarded maxillary growth (Alam et al., 2013), malposed teeth, crowding and rotation of teeth, and a high incidence of class III malocclusion (Haque and Alam, 2015).

Most of the previous studies relating to craniofacial anomalies have used 2D imaging, such as Late. Ceph. which was cost-effective with low radiation exposure and the study of various landmarks were done efficiently by linear and angular measurements (Alkofide, 2008). The morphology of ST can be efficiently measured with Late. Ceph. With the advancement in radiographic techniques and imaging, there is a shift toward 3D imaging techniques, particularly 3D imaging using CT scan (Hasan et al., 2016a,b, 2019; Islam et al., 2017) and CBCT (Yasa et al., 2017) as they give a better and accurate extent of the lesions in a 3D view and hence play a key role in the diagnosis and treatment of craniofacial malformations.

Extensive search of literature relating to the measurement of ST revealed that there was only one study on clefts in Saudi population with little or no focus on its relation to ST (Alkofide, 2008). Very few studies have evaluated the postnatal development and structure of ST and its relation to clefts (Alkofide, 2008; Yasa et al., 2017) which measured only three parameters to establish the morphology of ST. Due to limited research in this area and alarming number of individuals with clefts without the syndrome in Saudi Arabia with this genotype, the current investigation was undertaken to calculate the seven parameters of morphology of the ST, and to compare the findings with non-cleft (NC) healthy subjects with the following aims:

1. Investigation of ST bridging, type of skeletal malocclusion, and different dental anomalies.
2. Gender disparities of seven parameters of morphology of the ST among cleft and NC subjects.
3. Multiple comparisons of seven parameters of morphology of the ST among four different types of cleft and NC subjects.

## Materials and Methods

In this retrospective study, clinical and radiographic details of 31 NC subjects and 92 cleft subjects were used. All the records were collected from Saudi board Dental residents. The research protocol was prepared by one calibrated specialist orthodontist and the data were stored. The protocol was submitted for ethical board review. After approval, data investigations and analysis were completed. The details of ethical approval number are shown in Table 1. Out of 92 cleft subjects, 29 had BCLP, 41 had UCLP, nine had UCLA, and 13 had UCL as per cleft classification details from the clinical records. The details of age and gender distribution, demographic details, and inclusion and exclusion criteria are presented in Table 1.

**TABLE 1:** Demographic details and methods.

Population	Saudi subjects						
Inclusion and exclusion criteria	Non syndromic cleft subjects with good quality x-ray images. No history of craniofacial surgical treatment besides lip and palate surgery. No orthodontic treatment has been done. No anatomical variation in the ST and sphenoidal regions. Matched with healthy control without any craniofacial deformity. Subjects using hormonal medications or corticosteroids were excluded from the study.						
Sampling	Convenient sampling following inclusion and exclusion criteria.						

**Type of cleft**			**Non-cleft**	**BCLP**	**UCLP**	**UCLA**	**UCL**

Subjects distribution	Male		14	19	26	3	7
	Female		17	10	15	6	6
	Total (*N* = 123)		31	29	41	9	13
Age			13.29 ± 3.52	14.07 ± 4.73	14.32 ± 4.46	12.78 ± 4.09	13.31 ± 4.46
Data used	Digital lateral cephalogram, orthopantomogram, and clinical record details.						
Ethical clearance	Protocol has been presented to the ethical board of Alrass Dental Research Center, Qassim University. Ethical clearance has been obtained with the Code #: DRC/009FA/20.						

**Method**	**Artificial intelligence driven technique using Webceph software (Korea)**						

Landmarks used and the details	TS		Tuberculum sella	The most anterior point of the contour of the sella turcica			
	DS		Dorsum sellae	The posterior wall of the sella turcica			
	SF		Sella floor	The deepest point on the floor of pituitary fossa			
	Pclin		Posterior clenoid	The most anterior point of the PClin process			
	SA		Sella anterior	The most anterior point of the sella			
	SP		Sella posterior	The most posterior point of the sella			
	SM		Sella median	A point midway between PClin and TS			

**Measurements (seven parameters)**			**Significance/importance**				

a	Sella length	TS-Pclin	Changes in the size of sella turcica are often identified with the pathology of pituitary gland and may have an undetected hidden disease; hence, sella length is one of the parameters to determine the sella size.				
b	Sella width	SA-SP	Utilized clinically for pubertal growth phase determination, would be increased by advanced age, it has strong correlation with age.				
c	Sella diameter	TS-DS	Growth of an individual can be assessed based on the diameter of the sella turcica at different age periods.				
d	Sella height anterior	TS-SF	As the anterior part of the sella turcica is believed to develop mainly from neural crest cell, so we need to measure the sella height anterior. So that, we can assume or determine any structural deviation in the anterior wall which are believed to be associated with the specific deviation in the facial structure.				
e	Sella height posterior	PClin-SF	The posterior part of the sella turcica develops from the para-axial-mesoderm, which develops approximately 7 weeks of gestation. If any disturbance occurs in this area it remains throughout the life, as the time of formation of sella closely associated with the development of maxilla. So that, it may be assumed that any aberration leading to cleft may be associated with some fault at the level of sella turcica.				
f	Sella height median	SM-SF	Utilized clinically for pubertal growth phase determination, would be increased with age.				
g	Sella area	TS-SA-SF-SP-Pclin	During embryological development of the sella area is the key point for the migration of the neural crest cells to the fronto nasal and maxillary developmental fields. Pituitary fossa increased in size with age and found a positive correlation of the area of the sella to age.				

**FIGURE 1:**
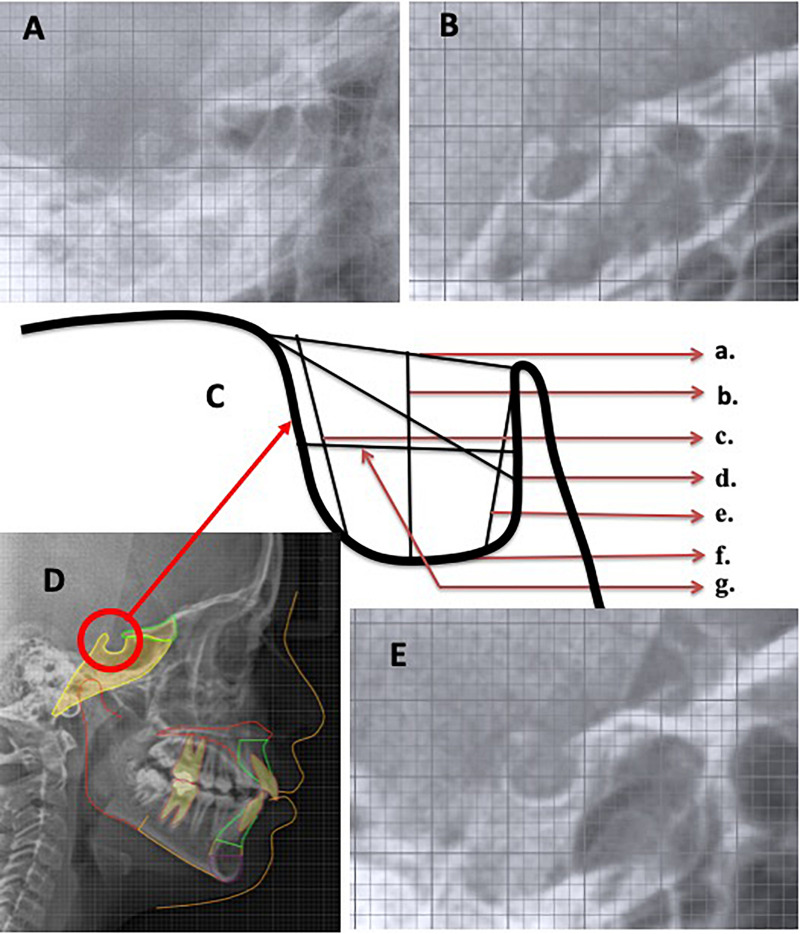
Lateral cephalogram view of sella turcica in **(A)** BCLP, **(B)** UCLP, **(C)** sella turcica parameters: **(a)** TS-Pclin; **(b)** SA-SP; **(c)** TS-DS; **(d)** TS-SF; **(e)** PClin-SF; **(f)** SM-SF; and **(g)** sella area, **(D)** Control, and **(E)** UCLA subjects.

Lateral cephalogram X-rays were used to investigate of ST bridging by two observers and the data were recorded after agreement by both the observers and analyzed. In a similar manner, each orthopantomogram (OPG) was investigated and dental anomalies are listed after agreement by both the observers in cases with ST bridging. Late. Ceph. X-ray was also used for skeletal class of malocclusion assessment (based on ANB and Wits measurement) only in cases with ST bridging and seven parameters of ST morphology in all subjects (Hasan et al., 2016a,b, 2019; Islam et al., 2017) were measured by one examiner using artificial intelligence driven Webceph software (Korea). The details of the seven parameters measurements are presented in Table 1 (Hasan et al., 2016a,b, 2019; Islam et al., 2017) and shown in Figure 1 (Hasan et al., 2016a,b, 2019; Islam et al., 2017).

### Statistical Analyses

After a 2-week interval, 20 randomly selected X-rays were used for re-measurement in a similar fashion. For ST bridging and dental anomalies results were tested using Kappa test for intra and inter-examiner reliability. Error testing in the investigation of ST morphology based on seven parameters measurements were tested by intra-class correlation co-efficient (ICC) test. Total investigated data were analyzed using version 26.0 SPSS software (IBM, Armonk, NY, United States). Normality of the measured seven parameters of ST morphology data was assessed using Kolmogorov–Smirnov test. Descriptive statistics were calculated for each parameter and presented in a tabulated format. Independent *t*-test was used for gender disparities and ANOVA test used for multiple comparison among NC and all four types of cleft groups.

## Results

Using Kolmogorov–Smirnov test, measured seven parameters of ST morphology data were found normally distributed. Error test results of the ST bridging and dental anomalies investigation showed excellent intra and inter-examiner reliability. ICC results for all seven parameters of ST morphology ranged from 0.86 to 0.94.

Prevalence of ST bridging, type of malocclusion involved, and associated dental anomalies are listed in Table 2. Overall, 6.45 and 22.82% ST bridging was found in NC and cleft individuals, respectively. Among four types of clefts, ST bridging found, UCL > UCLP > BCLP > UCLA. Highest % in UCL (30.77%). Skeletal Class III malocclusion was found to be more prevalent in ST bridging individuals. Among dental anomalies, impacted canine, congenital missing, and supernumerary teeth were found to be common.

**TABLE 2:** Sella turcica bridging, type of malocclusion and associated anomalies.

Gender	Sella bridging	Subject	Skeletal malocclusion	Dental anomalies	Prevalence
F	Complete	Non-cleft	Class I	IC	Non-cleft = 6.45%
M	Partial	Non-cleft	Class II	IC	
F	Partial	Bilateral Cleft Lip and Palate	Class III	CM + IC	BCLP = 20.69%
M	Partial	Bilateral Cleft Lip and Palate	Class III	CM	
M	Partial	Bilateral Cleft Lip and Palate	Class III	None	
M	Partial	Bilateral Cleft Lip and Palate	Class III	CM	
M	Partial	Bilateral Cleft Lip and Palate	Class III	CM + IC	
M	Partial	Bilateral Cleft Lip and Palate	Class III	None	
M	Partial	Unilateral Cleft Lip and Palate Lt Side	Class III	CM	UCLP = 24.39%
F	Partial	Unilateral Cleft Lip and Palate Lt Side	Class III	IC	
M	Partial	Unilateral Cleft Lip and Palate Lt Side	Class III	CM	
F	Complete	Unilateral Cleft Lip and Palate Lt Side	Class III	CM + IC	
F	Partial	Unilateral Cleft Lip and Palate Lt Side	Class III	CM + IC	
M	Partial	Unilateral Cleft Lip and Palate Lt Side	Class III	IC	
F	Complete	Unilateral Cleft Lip and Palate Lt Side	Class III	CM + IC + Dilaceration	
M	Partial	Unilateral Cleft Lip and Palate Rt Side	Class I	CM	
M	Partial	Unilateral Cleft Lip and Palate Rt Side	Class III	CM + IC	
M	Complete	Unilateral Cleft Lip and Palate Rt Side	Class I	CM	
F	Partial	Unilateral Cleft Lip and Alveolus Rt Side	Class III	CM	UCLA = 11.11%
F	Complete	Unilateral Cleft Lip Lt Side	Class III	CM	UCL = 30.77%
M	Partial	Unilateral Cleft Lip Rt Side	Class III	SN	
M	Partial	Unilateral Cleft Lip Rt Side	Class II	CM + IC	
F	Complete	Unilateral Cleft Lip Rt Side	Class III	CM	

*Non-cleft = M, male; F, female; IC, impacted canine; CM, congenital missing; SN, supernumerary tooth.*

Table 3 shows the details of descriptive and comparative gender disparities results among NC and different types of clefts (NC, BCLP, UCLP, UCLA, and UCL). Overall ST morphometry has been presented which shows no significant gender disparities.

**TABLE 3:** Gender disparities of all sella turcica morphomometric parameters among all five groups.

Group	Variables	Gender	Mean	SD	95% CI		*p*
					Lower	Upper	
Control	A	Male	11.164	1.690	−0.685	1.369	0.501
		Female	10.822	1.090			
	B	Male	9.669	1.466	−1.105	1.123	0.987
		Female	9.659	1.544			
	C	Male	10.925	3.296	−1.489	2.966	0.503
		Female	10.187	2.771			
	D	Male	8.210	1.461	−0.649	1.708	0.366
		Female	7.681	1.698			
	E	Male	7.779	0.911	−1.049	1.329	0.812
		Female	7.639	2.007			
	F	Male	8.746	1.131	−0.531	1.355	0.379
		Female	8.335	1.385			
	G	Male	77.133	22.446	−9.325	23.683	0.381
		Female	69.954	22.288			
BCLP	A	Male	8.075	0.989	−0.390	1.495	0.239
		Female	7.522	1.480			
	B	Male	7.422	1.289	−0.520	1.639	0.297
		Female	6.862	1.455			
	C	Male	7.663	2.258	−1.330	2.366	0.570
		Female	7.145	2.398			
	D	Male	6.254	1.487	−0.897	1.452	0.632
		Female	5.977	1.420			
	E	Male	6.229	1.142	−0.876	1.083	0.829
		Female	6.125	1.368			
	F	Male	7.210	1.308	−0.928	1.365	0.699
		Female	6.991	1.650			
	G	Male	49.767	17.188	−9.614	18.425	0.525
		Female	45.362	18.076			
UCLP	A	Male	8.223	1.266	−0.381	1.308	0.273
		Female	7.759	1.324			
	B	Male	7.774	1.492	−1.081	1.018	0.951
		Female	7.806	1.778			
	C	Male	9.392	2.418	−1.659	1.480	0.908
		Female	9.481	2.348			
	D	Male	6.903	1.227	−1.034	0.513	0.499
		Female	7.164	1.089			
	E	Male	6.895	1.054	−0.798	0.549	0.710
		Female	7.019	0.977			
	F	Male	7.278	1.158	−0.872	0.582	0.689
		Female	7.423	1.013			
	G	Male	57.560	12.685	−6.058	10.327	0.601
		Female	55.425	12.139			
UCL	A	Male	8.873	1.495	−0.940	2.356	0.365
		Female	8.165	1.141			
	B	Male	8.034	1.636	−1.968	2.016	0.979
		Female	8.010	1.616			
	C	Male	9.739	2.744	−3.412	2.480	0.734
		Female	10.205	1.923			
	D	Male	7.199	1.377	−2.404	0.968	0.369
		Female	7.917	1.378			
	E	Male	7.543	0.845	−1.474	1.400	0.956
		Female	7.580	1.474			
	F	Male	7.711	1.692	−2.626	1.652	0.626
		Female	8.198	1.810			
	G	Male	62.428	21.557	−29.800	19.872	0.669
		Female	67.392	18.638			
UCLA	A	Male	8.303	1.414	−2.153	1.033	0.433
		Female	8.863	0.686			
	B	Male	8.120	1.026	−3.044	2.150	0.696
		Female	8.567	1.719			
	C	Male	10.167	0.257	−3.081	4.514	0.669
		Female	9.450	2.683			
	D	Male	6.823	0.770	−2.662	2.129	0.800
		Female	7.090	1.624			
	E	Male	6.923	1.822	−3.178	3.048	0.962
		Female	6.988	1.878			
	F	Male	7.040	1.574	−3.876	2.599	0.655
		Female	7.678	2.064			
	G	Male	54.233	10.970	−39.066	31.380	0.804
		Female	58.076	23.940			

* p < 0.05 considered statistically significant.

Table 4 shows the description total details among all five groups (NC, BCLP, UCLP, UCLA, and UCL) subjects. Multiple comparison results are presented in Table 5. Significantly larger TS-Pclin has been found in NC group in comparison to all four-cleft group (*p* < 0.001). However, there are no significant differences within the cleft group found. Smallest value found in BCLP group was 7.884 mm. Sa-SP value shows significant disparities between NC vs BCLP (*p* < 0.001), NC vs UCLP (*p* < 0.001), and NC vs UCL (*p* = 0.012) groups. When TS-DS values were compared, NC vs BCLP (*p* < 0.001), BCLP vs UCLP (*p* = 0.018) and UCL (*p* = 0.037) showed significant disparities. There were significant disparities between NC vs BCLP (*p* < 0.001) and BCLP vs UCL (*p* = 0.019) in PClin-SF parameter. And, when compared the parameters of SM-SF and TS-SA-SF-SP-PClin, NC vs BCLP and NC vs UCLP shows significant disparities. In BCLP group, values of all seven parameters of ST morphometry showed smallest values in comparison with all four groups.

**TABLE 4:** Descriptive results of all sella turcica morphomometric parameters among all five groups.

	TS-PClin		SA-SP		TS-DS		TS-SF		PClin-SF		SM-SF		TS-SA-SF-SP-PClin	
	Mean	SD	Mean	SD	Mean	SD	Mean	SD	Mean	SD	Mean	SD	Mean	SD
Control	10.976	1.379	9.664	1.484	10.520	2.991	7.920	1.592	7.702	1.585	8.521	1.273	73.196	22.281
BCLP	7.884	1.185	7.229	1.350	7.484	2.278	6.159	1.445	6.193	1.201	7.134	1.409	48.248	17.306
UCLP	8.053	1.291	7.786	1.580	9.424	2.363	6.999	1.172	6.940	1.016	7.331	1.096	56.779	12.378
UCL	8.546	1.340	8.023	1.557	9.954	2.316	7.530	1.370	7.560	1.124	7.936	1.691	64.720	19.589
UCLA	8.677	0.934	8.418	1.470	9.689	2.155	7.001	1.347	6.967	1.742	7.466	1.839	56.795	19.799

**TABLE 5:** Multiple comparison of all sella turcica morphomometric parameters among all five groups.

Variables	Multiple comparison			MD	SE	95% CI		*p*-Value
						Lower bound	Upper bound	
TS-PClin	Control	vs	BCLP	3.09199*	0.329	2.150	4.034	0.000
			UCLP	2.92271*	0.303	2.055	3.790	0.000
			UCL	2.42998*	0.421	1.226	3.634	0.000
			UCLA	2.29946*	0.482	0.919	3.680	0.000
	BCLP	vs	UCLP	−0.16928	0.309	−1.054	0.715	1.000
			UCL	−0.66202	0.425	−1.879	0.555	1.000
			UCLA	−0.79253	0.486	−2.183	0.598	1.000
	UCLP	vs	UCL	−0.49274	0.406	−1.653	0.667	1.000
			UCLA	−0.62325	0.469	−1.965	0.718	1.000
	UCL	vs	UCLA	−0.13051	0.552	−1.711	1.450	1.000
SA-SP	Control	vs	BCLP	2.43493*	0.386	1.331	3.539	0.000
			UCLP	1.87769*	0.356	0.860	2.895	0.000
			UCL	1.64047*	0.494	0.228	3.053	0.012
			UCLA	1.24577	0.566	−0.373	2.864	0.296
	BCLP	vs	UCLP	−0.55723	0.363	−1.594	0.480	1.000
			UCL	−0.79446	0.499	−2.221	0.632	1.000
			UCLA	−1.18916	0.570	−2.820	0.442	0.391
	UCLP	vs	UCL	−0.23722	0.476	−1.598	1.123	1.000
			UCLA	−0.63192	0.550	−2.205	0.941	1.000
	UCL	vs	UCLA	−0.3947	0.648	−2.248	1.459	1.000
TS-DS	Control	vs	BCLP	3.03586*	0.646	1.187	4.885	0.000
			UCLP	1.09561	0.595	−0.608	2.799	0.683
			UCL	0.56615	0.827	−1.799	2.931	1.000
			UCLA	0.83111	0.947	−1.879	3.541	1.000
	BCLP	vs	UCLP	−1.94025*	0.607	−3.677	−0.204	0.018
			UCL	−2.46971*	0.835	−4.858	−0.081	0.037
			UCLA	−2.20475	0.955	−4.936	0.526	0.226
	UCLP	vs	UCL	−0.52946	0.796	−2.807	1.749	1.000
			UCLA	−0.2645	0.921	−2.899	2.370	1.000
	UCL	vs	UCLA	0.26496	1.085	−2.838	3.368	1.000
TS-SF	Control	vs	BCLP	1.76106*	0.358	0.737	2.785	0.000
			UCLP	0.92114	0.330	−0.022	1.864	0.061
			UCL	0.38968	0.458	−0.920	1.699	1.000
			UCLA	0.91857	0.525	−0.582	2.419	0.825
	BCLP	vs	UCLP	−0.83992	0.336	−1.801	0.122	0.138
			UCL	−1.37138*	0.462	−2.694	−0.049	0.037
			UCLA	−0.84249	0.529	−2.355	0.670	1.000
	UCLP	vs	UCL	−0.53146	0.441	−1.793	0.730	1.000
			UCLA	−0.00257	0.510	−1.461	1.456	1.000
	UCL	vs	UCLA	0.52889	0.601	−1.190	2.247	1.000
PClin-SF	Control	vs	BCLP	1.50883*	0.333	0.555	2.463	0.000
			UCLP	0.76169	0.307	−0.117	1.640	0.146
			UCL	0.14194	0.426	−1.078	1.362	1.000
			UCLA	0.73527	0.489	−0.663	2.133	1.000
	BCLP	vs	UCLP	−0.74714	0.313	−1.643	0.149	0.186
			UCL	−1.36690*	0.431	−2.599	−0.135	0.019
			UCLA	−0.77356	0.492	−2.182	0.635	1.000
	UCLP	vs	UCL	−0.61976	0.411	−1.795	0.555	1.000
			UCLA	−0.02642	0.475	−1.385	1.333	1.000
	UCL	vs	UCLA	0.59333	0.560	−1.008	2.194	1.000
SM-SF	Control	vs	BCLP	1.38651*	0.348	0.392	2.381	0.001
			UCLP	1.18991*	0.320	0.274	2.106	0.003
			UCL	0.58449	0.445	−0.688	1.857	1.000
			UCLA	1.05509	0.510	−0.403	2.513	0.406
	BCLP	vs	UCLP	−0.19659	0.327	−1.131	0.738	1.000
			UCL	−0.80202	0.449	−2.087	0.483	0.767
			UCLA	−0.33142	0.513	−1.800	1.138	1.000
	UCLP	vs	UCL	−0.60542	0.428	−1.831	0.620	1.000
			UCLA	−0.13482	0.495	−1.552	1.282	1.000
	UCL	vs	UCLA	0.4706	0.584	−1.199	2.140	1.000
TS-SA-SF-SP-PClin	Control	vs	BCLP	24.94809*	4.584	11.835	38.061	0.000
			UCLP	16.41760*	4.223	4.336	28.499	0.002
			UCL	8.4768	5.863	−8.295	25.249	1.000
			UCLA	16.40092	6.718	−2.819	35.621	0.161
	BCLP		UCLP	−8.53049	4.305	−20.847	3.786	0.499
			UCL	−16.4713	5.922	−33.414	0.471	0.063
			UCLA	−8.54716	6.770	−27.915	10.821	1.000
	UCLP		UCL	−7.9408	5.648	−24.097	8.216	1.000
			UCLA	−0.01667	6.531	−18.702	18.668	1.000
	UCL		UCLA	7.92413	7.694	−14.087	29.935	1.000

* p < 0.05 considered statistically significant.

## Discussion

Unique quality of this study is that five different groups of subjects were investigated. Only one study has been found based on literature search and used three groups of subjects of Saudi population. ST bridging, type of skeletal malocclusion, and associated dental anomalies at time in a single study have not been investigated before. All seven parameters of ST morphology (Hasan et al., 2016a,b, 2019; Islam et al., 2017) are investigated in this study. Previous studies measured three parameters of ST morphology and ST bridging only (Alkofide, 2008; Yasa et al., 2017).

A thorough knowledge of ST and its variations is very important to identify it from medically compromised patients such as spina bifida or craniofacial deviations (Axelsson et al., 2004). In a study by Alkofide (2008), the morphological variations of ST were assessed in CLP patients and it was found that most of the patients had morphological deviations such as irregular posterior wall and double contour of the floor as compared to normally formed ST. Second, in the NC subjects included in the study, the morphology of ST was normal as compared to the people with clefts. In the earlier study, it was shown that ST bridging was 5.5–22% in normal person, while it was 6.45% in the NC individuals. In the present study, it is 22.82% overall in the cleft patients. However, its occurrence was more in patients with craniofacial deviations. ST bridging was 30.77% in subjects with UCL in the present study. Under such circumstances, it draws attention and marks the direction for future research and study if ST bridge exists in normal individuals in the current population.

Various investigations have been done on the morphology of ST with varying techniques (Axelsson et al., 2004; Alkofide, 2008; Hasan et al., 2016a,b, 2019; Islam et al., 2017; Yasa et al., 2017). In the current study, no significant gender disparities of the ST morphology in all seven parameters were found. Taking into account the results of the current and the previous studies (Islam et al., 2017; Yasa et al., 2017), gender disparities were measurably insignificant for all linear and area measurements of ST. According to Weisberg et al. (1976), individuals with abnormal ST may suffer from undetected hidden disease. Hence, from an altered state of ST, pathology or anomaly can be identified that may influence the secretion of hormones such as growth hormone, prolactin, follicle stimulating hormone, and thyroid stimulating hormone (Alkofide, 2007).

The results revealed significant disparities in different parameters of the ST morphology in cleft subjects (BCLP, UCLP, UCLA, and UCL) as compared to the NC and also among different types of cleft subjects (BCLP, UCLP, UCLA, and UCL). BCLP subjects exhibited smaller measurements in all parameters compared to the other groups. Results revealed disparities in the measured three parameters of ST morphology are smaller (Alkofide, 2008) and larger (Yasa et al., 2017) between cleft subjects than in NC subjects. Alkofide (2008) found smaller measurements in UCLP subjects. Yasa at al. found larger values in all three measured parameters in cleft group, only length showed highly significant disparities, however, the type of cleft was not mentioned. In another study, data of 62 subjects with palatally impacted canine revealed significant disparities in ST bridging and three parameters of ST morphology as compared to the control in Saudi population (Baidas et al., 2018).

Studies in the past have shown that patients with disorders or syndromes such as holoprosencephaly (Kjær et al., 2002), Down syndrome (Hasan et al., 2019), spina bifida (Kjær et al., 1999), CLP (Alkofide, 2008; Yasa et al., 2017), fragile X syndrome (Kjær et al., 2001), Williams syndrome (Axelsson et al., 2004), and severe craniofacial deformities (Becktor et al., 2000) have craniofacial malformations which affect the size and/or morphology of ST.

It is well established that the anatomy of ST is variable, and it is of remarkable importance in orthodontics. The anterior form of ST may aid in predicting the patient growth and in surveying craniofacial morphology (Bishara and Athanasiou, 1995). An orthodontist should be aware of the normal variations in the ST which might help in identifying any pathology associated with it (Du Boulay and Trickey, 1967). The outcomes suggest that ST bridging and altered ST morphology in CLP subjects required careful monitoring of skeletal malocclusion, dental anomalies, and canine eruption are required to diagnosed and guide for better management at an early age.

## Conclusion

ST bridging, type of skeletal malocclusion, and associated dental anomalies are common in cleft subjects compared to NC subjects. No significant gender disparities were found in four different types of cleft vs NC subjects. All seven parameters of ST morphology are smaller in NC subjects compared to those with clefts. BCLP subjects had smaller measurements in all seven parameters of ST morphology as compared to NC and all other types of cleft subjects.

## Data Availability Statement

All datasets presented in this study are included in the article/Supplementary Material.

## Ethics Statement

The studies involving human participants were reviewed and approved by the Ethical Board of Alrass Dental Research Center, Qassim University. Ethical clearance has been obtained with the Code #: DRC/009FA/20. Written informed consent to participate in this study was provided by the participants’ legal guardian/next of kin.

## Author Contributions

Both authors contributed to the article and approved the submitted version.

## Conflict of Interest

The authors declare that the research was conducted in the absence of any commercial or financial relationships that could be construed as a potential conflict of interest.
