# Supplementary material for: Evaluation of Sella Turcica Bridging and Morphology in Different Types of Cleft Patients
Source: Front Cell Dev Biol. 2020 Jul 22;8:656. doi: 10.3389/fcell.2020.00656 (PMC7387404; doi:10.3389/fcell.2020.00656)
Supplement: Supplementary file 1 [file Data_Sheet_1.PDF]

Skip to Main Content

Cleft ST

by Ravi 1

FILE	ST_CLEFT_MANUSCRIPT_PLG_CHK.DOCX (20.86K)		
TIME SUBMITTED	29-APR-2020 12:26PM (UTC+0000)	WORD COUNT	2358
SUBMISSION ID	1303531533	CHARACTER COUNT	12777

Sella Turcica bridging and 7 different morphologies in 4 different types of cleft versus non-cleft subjects.

ABSTRACT

Objectives: To evaluate sella turcica (ST) bridging, associated anomalies and morphology, in subjects with 4 different type of clefts, and compare them with non-cleft subjects.

Material and Methods: A total of 123 (31 non-cleft and 92 cleft) Saudi subjects who had their lateral cephalogram (Late. Ceph.), orthopantomogram (OPG) and clinical details for ordinary diagnosis were included in the study. Among 92 cleft subjects, 29 had bilateral cleft lip and palate (BCLP), 41 had Unilateral cleft lip and palate (UCLP), 9 had unilateral cleft lip and alveolus (UCLA) and 13 with unilateral cleft lip (UCL). ST bridging and 7 parameters related to ST morphology and skeletal malocclusion was analysed using Late. Ceph. Associated dental anomalies in ST bridging subjects were investigated using OPG. The images were investigated using artificial intelligence driven Webceph software. Multiple statistical tests were applied to see the differences between gender and among cleft versus non-cleft subjects.

Results: ST bridging was found to be higher in cleft subjects (22.82%). Most of the cleft subjects had severe skeletal Class III malocclusion associated with multiple types of dental anomalies (impacted canines, congenital missing and presence of supernumerary teeth). No significant gender disparities in all 7 parameters of ST morphology were found between non-cleft and cleft groups. However, there were significant differences when compared among 4 different types of cleft individuals versus non-cleft subjects.

Conclusion: ST bridging is more prevalent in cleft subjects along with Class III malocclusion and associated dental anomalies. ST morphometry differs significantly between cleft versus non cleft subjects. Bilateral cleft lip and palate subjects (BCLP) exhibits smaller values of all 7 parameters as compared to all other groups.

Keywords: Sella turcica; sella turcica bridging; morphometry; bilateral cleft lip and palate; unilateral cleft lip and palate.

INTRODUCTION

Late. Ceph uses a number of landmarks as reference points for analysis/study of craniofacial structures. ST serves as one such important landmark in the cranium on Late. Ceph. The sella point or the centre of the ST is a point in the cranial base which is situated at the midpoint of ST that accommodates the pituitary gland (Celik-Karatas, 2015). It plays an important role in cephalometric analysis and helps us identify pathologies related to pituitary gland and hence becomes an exceptional source of information, specifically those syndromes that affect craniofacial region. A thorough knowledge of its radiological anatomy and variations may help us evaluate the growth and recognize any deviation in a variety of anomalies or pathological situations, and the possible outcome of the orthodontic treatment in such situations.

Congenital anomalies, though identified at birth often, get initiated during pregnancy due to chromosomal abnormalities. A gamut of congenital anomalies occur in the craniofacial region, cleft lip and palate (CLP) being the most common anomaly in the head and neck region, only second to congenital heart disease in the whole body. Hence, cleft deformities have been included in their Global Burden of Disease initiative, by World Health Organization (WHO). CLP is quite variable in its presentation and affects about 1.17/1000 birth overall 1.30 of every 1000 live births in Saudi (Sabbag et al. 2015) and Asian populations (Cooper et al., 2006). CLP has a multifactorial etiology with genetics and environmental factors to be the major contributing factors (Mars and Houston et al., 1990). The clefts have been classified depending upon the extent of involvement and their location as cleft palate, cleft lip, unilateral cleft lip (UCL), unilateral cleft lip and alveolus (UCLA), unilateral cleft lip and palate (UCLP) and bilateral cleft lip and palate (BCLP) etc. The affected children may have retarded maxillary growth (Alam et al. 2013), malposed teeth, crowding and rotation of teeth, and a high incidence of class III malocclusion (Haque and Alam 2015).

Most of the previous studies relating to craniofacial anomalies have used 2D imaging, such as Late. Ceph. which was cost-effective with low radiation exposure and the study of various landmarks were done efficiently by linear and angular measurements (Alkofide 2008). The morphology of ST can be efficiently measured with Late. Ceph. However, with the advancement in radiographic techniques and imaging, there is a shift towards 3D imaging techniques, particularly 3D imaging using CT scan (Hasan et al. 2016a, 2016b, 2019; Islam et al. 2017) and CBCT (Yasa et al. 2017) as they give a better and accurate extent of the lesions and hence play a key role in the diagnosis and treatment of craniofacial malformations.

Extensive search of literature relating to the measurement of ST revealed that there was only one study on clefts in Saudi population with little or no focus on its relation to ST (Alkofide 2008). Very few studies have evaluated the postnatal development and structure of ST and its relation to clefts (Alkofide 2008; Yasa et al. 2017) which measured only 3 parameters to establish the morphology of ST. Due to limited research in this area and alarming number of individuals with clefts without the syndrome in Saudi Arabia with this genotype, the current investigation was undertaken to calculate the 7 parameters of morphology of the ST, and to compare the findings with non-cleft healthy subjects with the following aims:

1. Investigation of ST bridging, type of skeletal malocclusion and different dental anomalies.
2. Gender disparities of 7 parameters of morphology of the ST among cleft and non-cleft subjects.
3. Multiple comparisons of 7 parameters of morphology of the ST among 4 different types of cleft and non-cleft subjects.

MATERIALS AND METHODS

In this retrospective study, clinical and radiographic details of 31 non-cleft subjects and 92 cleft subjects were used. All the records were collected from Saudi board clinical residents. The research protocol was prepared by one calibrated specialist orthodontist and the data was stored. The protocol was submitted for ethical board review. After approval, data investigations and analysis were completed. The details of ethical approval number are shown in table 1. Out of 92 cleft subjects, 29 had BCLP, 41 had UCLP, 9 had UCLA and 13 had UCL as per cleft classification details from the clinical records. The details of age and gender distribution, demographic details, inclusion and exclusion criteria are presented in the table 1.

Late. Ceph. X-rays were used to investigate of ST bridging by two observers and the data was recorded after agreement by both the observers and analyzed. In a similar manner each OPG was investigated and dental anomalies are listed after agreement by both the observers in cases with ST bridging. Late. Ceph. X-ray was also used for skeletal class of malocclusion assessment (based on ANB and Wits measurement) only in cases with ST bridging and 7 parameters of ST morphology (Hasan et al. 2016a, 2016b, 2019; Islam et al. 2017) were measured by one examiner using artificial intelligence driven Webceph software (Korea). The details of the 7 parameters measurements are presented in table 1 (Hasan et al. 2016a, 2016b, 2019; Islam et al. 2017) and shown in figure 1 (Hasan et al. 2016a, 2016b, 2019; Islam et al. 2017).

Statistical analyses

After a 2 week interval, 20 randomly selected x-rays were used for re-measurement in a similar fashion. For ST bridging and dental anomalies results were tested using Kappa test for intra and inter-examiner reliability. Error testing in the investigation of ST morphology based on 7 parameters measurements were tested by intra-class correlation co-efficient (ICC) test. Total investigated data was analysed using version 26.0 SPSS software (IBM, Armonk, NY, USA). Descriptive statistics were calculated for each parameter and presented in a tabulated format.

Independent t -test was used for gender disparities and ANOVA test used for multiple comparison among non-cleft and all 4 types of cleft group.

RESULTS

Error test results of the ST bridging and dental anomalies investigation showed excellent intra and inter-examiner reliability. ICC results for all 7 parameters of ST morphology ranged from 0.86 to 0.94.

Prevalence of ST bridging, type of malocclusion involved and associated dental anomalies are listed in the Table 2. Overall, 6.45% and 22.82% ST bridging was found in non-cleft and cleft individuals respectively. Among 4 types of clefts, ST bridging found, UCL>UCLP>BCLP>UCLA. Highest % in UCL (30.77%). Skeletal Class III malocclusion was found to be more prevalent in ST bridging individuals. Among dental anomalies, impacted canine, congenital missing and supernumerary teeth were found to be common.

Table 3 shows the details of descriptive and comparative gender disparities results among control and different types of clefts (BCLP, UCLP, UCLP and UCL). Overall ST morphometry has been presented which shows no significant gender disparities.

Table 4 shows the description total details among all 5 groups (control, BCLP, UCLP, UCLP and UCL) subjects. Multiple comparison results are presented in the Table 5. Significantly larger TS-Pclin has been found in control group in comparison to all 4 cleft group ($p<0.001$). However, there are no significant differences within the cleft group. Smallest value found in BCLP group was 7.884mm. Sa-SP value shows significant disparities between control vs BCLP ($p<0.001$), control vs UCLP ($p<0.001$) and control vs UCL ($p=0.012$) groups. When TS-DS values were compared, control vs BCLP ($p<0.001$), BCLP vs UCLP ($p=0.018$) and UCL ($p=0.037$) showed significant disparities. There were significant disparities between control vs BCLP ($p<0.001$) and BCLP vs UCL ($p=0.019$) in PClin-SF parameter. And, when compared the parameters of SM-SF and TS-SA-SF-SP-PClin, control vs BCLP and control vs

UCLP shows significant disparities. In BCLP group, values of all 7 parameters of ST morphometry showed smallest values in comparison with all 4 groups.

DISCUSSION

Unique quality of this study is, 5 different groups of subjects were investigated. Only 1 study has been found based on literature search, and used 3 groups of subjects of Saudi population. ST bridging, type of skeletal malocclusion and associated dental anomalies at time in a single study has not been investigated before. All 7 parameters of ST morphology (Hasan et al. 2016a, 2016b, 2019; Islam et al. 2017) are investigated in this study. Previous studies measured 3 parameters of ST morphology and ST bridging only (Alkofide 2008; Yasa et al. 2017).

A thorough knowledge of ST and its variations is very important to identify it from medically compromised patients such as spina bifida or craniofacial deviations (Axelsson et al. 2004). In a study by Alkofide (2008), the morphological variations of ST were assessed in CLP patients and it was found that most of the patients had morphological deviations such as irregular posterior wall and double contour of the floor as compared to normally formed ST. Secondly, in the non-cleft subjects included in the study, the morphology of ST was normal as compared to the people with clefts. In the earlier study, it was shown that ST bridging was 5.5-22% in normal person, while it was 6.45% in the non-cleft individuals. In the present study, it is 22.82% overall in the cleft patients. However, its occurrence was more in patients with craniofacial deviations. ST bridging was 30.77% in subjects with UCL in the present study. Under such circumstances, it draws attention and marks the direction for future research and study if ST bridge exists in normal individuals in the current population.

Various investigations have been done on the morphology of ST with varying techniques (Axelsson et al., 2004; Alkofide, 2008; Hasan et al., 2016a; Hasan et al., 2016b; Islam et al. 2017; Yasa et al. 2017; Hasan et al., 2019). In the current study, no significant gender

disparities of the ST morphology in all 7 parameters was found. Taking into account the results of the current and the previous studies (Islam et al. 2017; Yasa et al. 2017), gender disparities were measurably insignificant for all linear and area measurements of ST. According to Weisberg et al., (1976), individuals with abnormal ST may suffer from undetected hidden disease. Hence, from an altered state of ST, pathology or anomaly can be identified that may influence the secretion of hormones such as growth hormone, prolactin, follicle stimulating hormone and thyroid stimulating hormone (Alkofide, 2007)

The results revealed significant disparities in different parameters of the ST morphology in cleft subjects (BCLP, UCLP, UCLA and UCL) as compared to the non-cleft and also among different types of cleft subjects (BCLP, UCLP, UCLA and UCL). BCLP subjects exhibited smaller measurements in all parameters compared to the other groups. Results revealed disparities in the measured 3 parameters of ST morphology are smaller (Alkofide 2008) and larger (Yasa et al. 2017) between cleft subjects than in non-cleft subjects. Alkofide (2008) found smaller measurements in UCLP subjects. Yasa at al. found larger values in all 3 measured parameters in cleft group, only length showed highly significant disparities, however, the type of cleft was not mentioned. In an another study, data of 62 subjects with palatally impacted canine revealed significant disparities in ST bridging and 3 parameters of ST morphology as compared to the control in Saudi population (Baidas et al. 2017).

Studies in the past have shown that patients with disorders or syndromes such as holoprosencephaly (Kjær et al. 2002), Down syndrome (Haider et al. 2019), spina bifida (Kjær et al. 1999), cleft lip and palate (Alkofide 2008; Yasa et al. 2017), fragile X syndrome (Kjær et al. 2001), Williams syndrome (Axelsson et al. 2004), and severe craniofacial deformities (Becktor et al. 2000), have craniofacial malformations which affect the size and/or morphology of ST. It is well established that the anatomy of ST is variable and it is of remarkable importance in orthodontics. The anterior form of ST may aid in predicting the patient growth

and in surveying craniofacial morphology (Bishara and Athanasiou, 1995). An orthodontist should be aware of the normal variations in the ST which might help in identifying any pathology associated with it (Du Boulay and Trickey, 1967).

CONCLUSION

ST bridging, type of skeletal malocclusion and associated dental anomalies are common in cleft subjects compared to non-cleft subjects. No significant gender disparities were found in 4 different types of cleft versus non-cleft subjects. All 7 parameters of ST morphology are smaller in non-cleft subjects compared to those with clefts. BCLP subjects had smaller measurements in all 7 parameters of ST morphology as compared to non-cleft and all other type of cleft subjects.

Cleft ST

ORIGINALITY REPORT

%11	%3	%7	%7
SIMILARITY INDEX	INTERNET SOURCES	PUBLICATIONS	STUDENT PAPERS

PRIMARY SOURCES

1	Submitted to Universiti Sains Malaysia<br>Student Paper	%3
2	Yue Yue, Jinfeng Chen, Lei Bao, Junjie Wang, Yanwei Li, Qingzhu Zhang. "Fluoroacetate dehalogenase catalyzed dehalogenation of halogenated carboxylic acids: A QM/MM approach", Chemosphere, 2020<br>Publication	%2
3	worldwidescience.org<br>Internet Source	%1
4	Izadi, N., and P.E. Haers. "An investigation into the variables associated with length of hospital stay related to primary cleft lip and palate surgery and alveolar bone grafting", International Journal of Oral and Maxillofacial Surgery, 2012.<br>Publication	%1
5	Isaac Myara, Catherine Alamowitch, Odile Michel, Didier Heudes, Jean Bariety, Bernard Guy-Grand, Jacques Chevalier. "Lipoprotein	%1

Oxidation and Plasma Vitamin E in Nondiabetic Normotensive Obese Patients", Obesity Research, 2003
Publication

6	academic.oup.com<br>Internet Source	%1
7	"Cleft Lip and Palate", Springer Science and Business Media LLC, 2013<br>Publication	%1
8	era.library.ualberta.ca<br>Internet Source	<%1
9	Submitted to University of Florida<br>Student Paper	<%1
10	Shobha Sundareswaran, Ravisankar Vijayan, Praveen Santhakumaran Nair, Latheef Vadakkepediyakkal, Sreehari Sathyanadhan. "Cephalometric Appraisal of the Sella Turcica- A Literature Review", Iranian Journal of Orthodontics, 2019<br>Publication	<%1
11	Submitted to University of Sheffield<br>Student Paper	<%1
12	"Abstracts of Lectures and Scientific Posters", The European Journal of Orthodontics, 2016<br>Publication	<%1

Tara L. Whitehill, Cynthia H.-F. Chau. "Single-

13	word intelligibility in speakers with repaired cleft palate", Clinical Linguistics & Phonetics, 2009<br>Publication		<1%
14	Eman A. Alkofide. "Sella Turcica Morphology and Dimensions in Cleft Subjects", The Cleft Palate-Craniofacial Journal, 2008<br>Publication		<1%
15	Jaana Laitinen, Reijo Ranta, Joonas. "The association between dental arch dimensions and occurrence of Finnish dental consonant misarticulations in cleft lip/palate children", Acta Odontologica Scandinavica, 2009<br>Publication		<1%

EXCLUDE QUOTES	OFF	EXCLUDE MATCHES	OFF
EXCLUDE BIBLIOGRAPHY	ON		
